# Assessment of the effectiveness of the BOPPPS model combined with case-based learning on nursing residency education for newly recruited nurses in China: a mixed methods study

**DOI:** 10.1186/s12909-024-05202-x

**Published:** 2024-03-01

**Authors:** Yongli Wang, Yiqian Chen, Ling Wang, Wen Wang, Xiangyan Kong, Xiaodan Li

**Affiliations:** 1 Peking University People’s Hospital, No. 11 Xizhimen South Street, Xicheng Dist, 100044 Beijing, China; 2 Nursing School of Peking University, 100191 Beijing, China

**Keywords:** New nurses, BOPPPS model, Case‑based learning, Core competence, Mixed methods

## Abstract

**Background:**

Expanding new nurse training and education is a priority for nursing educators as well as a critical initiative to stabilize the nursing workforce. Given that there is currently no standardized program for the training of new nurses in China, we investigated the effectiveness of the bridge-in, objective, pre-assessment, participatory learning, post-assessment, and summary model combined with case-based learning ((BOPPPS-CBL) for the standardized training of new nurses.

**Methods:**

The mixed method approach with explanatory sequential (quantitative-qualitative) method was used. A questionnaire was used to compare the impact of the BOPPPS-CBL model and the Traditional Learning Model (TLM) on the core competencies of 185 new nurses for two years of standardized training. Quantitative data were analyzed using SPSS 22.0. Focus group interviews were used with four groups of new nurses and perceptions of BOPPPS-CBL training were recorded. Qualitative data were analyzed thematically.

**Results:**

According to the quantitative data, more new nurses agreed that the BOPPPS-CBL model stimulated their learning and improved their core nursing competencies than the TLM. The BOPPPS-CBL group outperformed the TLM group on theoretical knowledge tests. Qualitative data revealed that 87.5% of new nurses agreed on the value of BOPPPS-CBL training, and three themes were extracted: (1) role promotion; (2) formation of new thinking to solve clinical problems; and (3) suggestions for improvement.

**Conclusion:**

BOPPPS-CBL training had a significant impact on improving new nurses’ core competencies and promoting the transition of new nurses to clinical practice nurses in China. The study recommends BOPPPS-CBL training as an effective teaching model for the standardized training and education of new nurses.

**Supplementary Information:**

The online version contains supplementary material available at 10.1186/s12909-024-05202-x.

## Background

At the end of 2020, there were nearly 4.45 million registered nurses in China [1], and some studies predict that by 2035 [1, 2], the demand for nurses in China will be 6.75 per 1,000 people. Because of this, many nurses will enter the clinic in the future. Unfortunately, however, new nurses are one of the groups with a high incidence of adverse nursing events [3]. Research has shown that strengthening nursing education and training to improve core competencies at all stages of care can ensure patient safety and improve global health [4, 5]. New nurse training is a vital aspect of hospital nurse training and can help new nurses solve problems during the transition from the nursing student stage to the clinical nurse role.

New nurses encounter various obstacles [6], such as, in the nursing student stage, they focus on acquiring theoretical knowledge and lack nursing practical ability which together with the lack of clinical work experience, leads to a weak link between their theoretical knowledge and clinical practice. Moreover, due to the poor core competence of new nurses, when facing clinical work, they become mentally stressed with negative emotions such as fear, anxiety, and even burnout [7]. These factors often make it difficult for new nurses to feel professionally fulfilled, which can severely affect their clinical performance and career planning. As a result, some new nurses may decide to leave this field [8]. According to research, new nurses can successfully transition into the role of clinical practice nurses by strengthening their core competencies [9].

There are no uniform standards for nursing core competencies globally. The International Council of Nurses (ICN) states that nursing core competencies are the application of a nurse’s knowledge, skills, judgment, and personal attributes in the performance of nursing duties [10]. Nursing core competencies have been defined differently depending on the state of nursing in each country. The Australian Nurses and Midwives Association [11] considers nursing core competencies as the foundation of nursing practice and the criteria and basis for assessing nurses’ competence in the workplace, which encompasses skills, knowledge, attitudes, values, and competencies in the professional domain. In China, core competencies are defined as “knowledge, skills, attitudes, judgments, and clinical problem-solving abilities within the prescribed practical roles and environments“ [12]. In the UK, new nurses receive no less than four months of training with a focus on mentoring support [13]. Australia launched a one-year general practice training program for new graduate nurses to emphasize the importance of primary health care [14].

The National Health Commission of China issued the “Training Outline for Newly Recruited Nurses” (hereafter referred to as the “Outline”), which is a guideline for on-the-job training and continuing education. All new registered nurses (first-time job holders, regardless of education) must receive 2 years of clinical training in their nursing specialization upon entry, shortening the transition of new nurses to clinical practice nurses [15, 16]. However, the Outline is only a guiding document for in-service training and continuing education, and there is not yet a unified, specific, detailed, and standardized training system for new nurses nationwide. Most hospitals design their training system under the guidance of the Outline, whose effectiveness is yet to be considered.

Most hospital’s training content includes theoretical and skill training, the traditional learning model education method is that the teacher mainly explains, and nurses only passively accept this knowledge, not including some active teaching methods and techniques [17]. Furthermore, overall training takes a long time, and the training format is relatively simple, which does not encourage participation and enthusiasm, resulting in an unsatisfactory training effect [18]. The new-nurse education includes many knowledge points that focus on the ability to combine theory and practice [8]. Improving nurses’ core competencies during their 2-year standardized training and education is an urgent issue that must be addressed.

The bridge-in, objective, pre-assessment, participatory learning, post-assessment, and summary (BOPPPS) model is a closed-loop teaching process that emphasizes student participation and feedback and is internationally recognized for its effectiveness [19]. It is based on constructivist and humanistic learning theories. Based on the “student-centered” approach, the BOPPPS model divides the teaching design process into six links: bridge-in, objective, pre-assessment, participatory learning, post-assessment, and summary to ensure that the teaching objectives are met. Research confirms that the BOPPPS model has significant advantages over traditional teaching modes and places a greater emphasis on student participation [20]. The BOPPPS teaching model has been promoted and used in many nations worldwide. Shih [21] implemented the concept in a flipped classroom for a business etiquette course using quasi-experimental research, which resulted in increased teacher-student interaction, a more dynamic and fascinating class, and enhanced student learning outcomes (*t* = 3.10, *P* < 0.01). Zhen employed a mixed research design to investigate the usage of design ideas based on the BOPPPS model in his teaching practice, which enhanced teaching methods, raised student interest in learning from 65 to 90%, and improved students’ higher-order thinking [22]. Other studies have demonstrated how the BOPPPS model can enhance ophthalmology teaching [23] and dental materials education [24] by encouraging clinical thinking abilities. As a result, it is important to acknowledge the BOPPPS model’s usefulness in medical education.

Moreover, a recent systematic evaluation [25] revealed that case-based learning (CBL), constructed upon authentic contexts within a constructivist framework, proved to be a advantageous teaching strategy for improving the performance and case-analysis abilities of medical students. In bridging the knowledge gap between theoretical understanding and clinical practice, CBL disseminates knowledge through clinical cases, with students taking a central role and cases providing guidance [26, 27]. Additionally, it has been shown to enhance students’ problem-solving skills, critical thinking, and motivation to study [28].

In recent years, the teaching mode of BOPPPS combined with CBL has emerged, and it has been widely used in medical education such as continuing medical education and ophthalmology, among others, with satisfactory results [23, 29]. Our study used an explanatory mixed methods research design which employed a quantitative quasi-experimental comparative design and a qualitative descriptive design from nurse focus group interview analysis. The study’s objectives were as follows:

1. Investigate the effectiveness of the BOPPPS-CBL model in the training of new nurses in China.
2. Explore nurses’ experience and suggestions regarding BOPPPS-CBL training to improve the program.

## Methods

### Design

An interpretive sequential approach (quantitative-qualitative) mixed methods was used in this study [30]. Phase 1 was a quantitative experimental study, and Phase 2 was a descriptive qualitative study.

### Participants

Participants included new nurses with standardized training at Peking University People’s Hospital from September 2017 to August 2021. We used a convenience sampling to enroll 115 nurses in the traditional learning model group (TLM group) who were almost all new in 2017 (due to resignation, 2 of the 117 nurses were excluded) and 70 in the BOPPPS-CBL group who were about all new in 2019 (3 nurses out of 73 withdrew from the study midway due to sick leave; Table 1). Inclusion criteria were: registered nurses, nurses just recruited and having participated in standardized training who agreed to participate in the research, and signed informed consent. The exclusion criteria were those who could not complete all the research contents for resignation, leave, and other reasons.

**Table 1:** Course modules and training periods

	The first year of training	Learning Time(h)	The second year of training	Learning Time(h)
The first quarter (August-October)	Laws and Regulations	3.5	Nursing management of pressure ulcers and incontinence	4
Second quarter (November-January)	Intravenous therapy and blood specimen collection	3	Thrombosis prevention and management	3.5
The third quarter (February-April)	Fall and bed fall prevention and care	3.5	Abnormal ECG identification and treatment	3.5
Fourth quarter (May-July)	Management of acute and critically ill patients	4	Pharmaceutical Safety	3

### Training design

#### TLM group

The training program was developed under the requirements of the Chinese Nursing Regulations and the “Outline”. Basic theoretical knowledge training, clinical nursing operation technique training, and professional theory and practical ability training in rotating clinical departments comprised the training content. The training period was set at 2 years. The assessment methods chosen were theoretical examination and clinical practice ability assessment.

#### BOPPPS-CBL group

This group was built on the foundation of the TLM group using the combined BOPPPS model and CBL.

First, we set up a research team. Team members comprised a deputy director of the nursing department (*n* = 1), senior specialist nurses (*n* = 3), as well as clinical teaching management nurses (*n* = 4). The team leader was assigned as the nursing department deputy director in charge of the design and quality control of CBL programs. Senior specialist nurses were in charge of retrieving, sorting, writing, and implementing CBL programs, while clinical teaching management nurses handled program implementation and evaluation.

Second, according to the bridge-in of the BOPPPS model, the principles of CBL writing were formulated: (1) typicality: inclusion of typical cases within the specialty; (2) authenticity:taken from real cases; (3) relevance: ensuring a significant correlation between the cases and teaching objectives; (4) guidance: embedding questions in the cases to steer nurses in their case discussions, analysis, and stimulate their thought processes; and (5) difficulty: introducing cases with a certain level of complexity to foster the cultivation of nurses’ independent thinking and judgment.

Third, we established CBL training objectives based on the BOPPPS model objectives. The first draft of the training plan was created using a literature review and teaching materials, following the compilation principles.

Fourth, the implementation and evaluation of the CBL training plan were formulated using the BOPPPS model’s pre-assessment, post-assessment, participatory learning, and summary, i.e., an eight-session training program that was conducted every 3 months for 3 ∼ 4 h for 24 months. Before the case presentation, new nurses were given questions and quizzes to help them understand their existing knowledge structure and help them adjust their teaching content and methods. Subsequently, nurses participated in a full group discussion of the case. Following that, they were asked to list the nursing problems in the case while proposing the group’s solution strategies through problem discussion, as well as the group members’ contributions and sharing of insights to test the new nurses’ learning and assess their overall ability to participate in discussions, communicate, ask questions, and solve problems. Finally, the teacher provided feedback, summarized the case study’s key points, and guided the new nurses in their reflection.

**Fig. 1:**
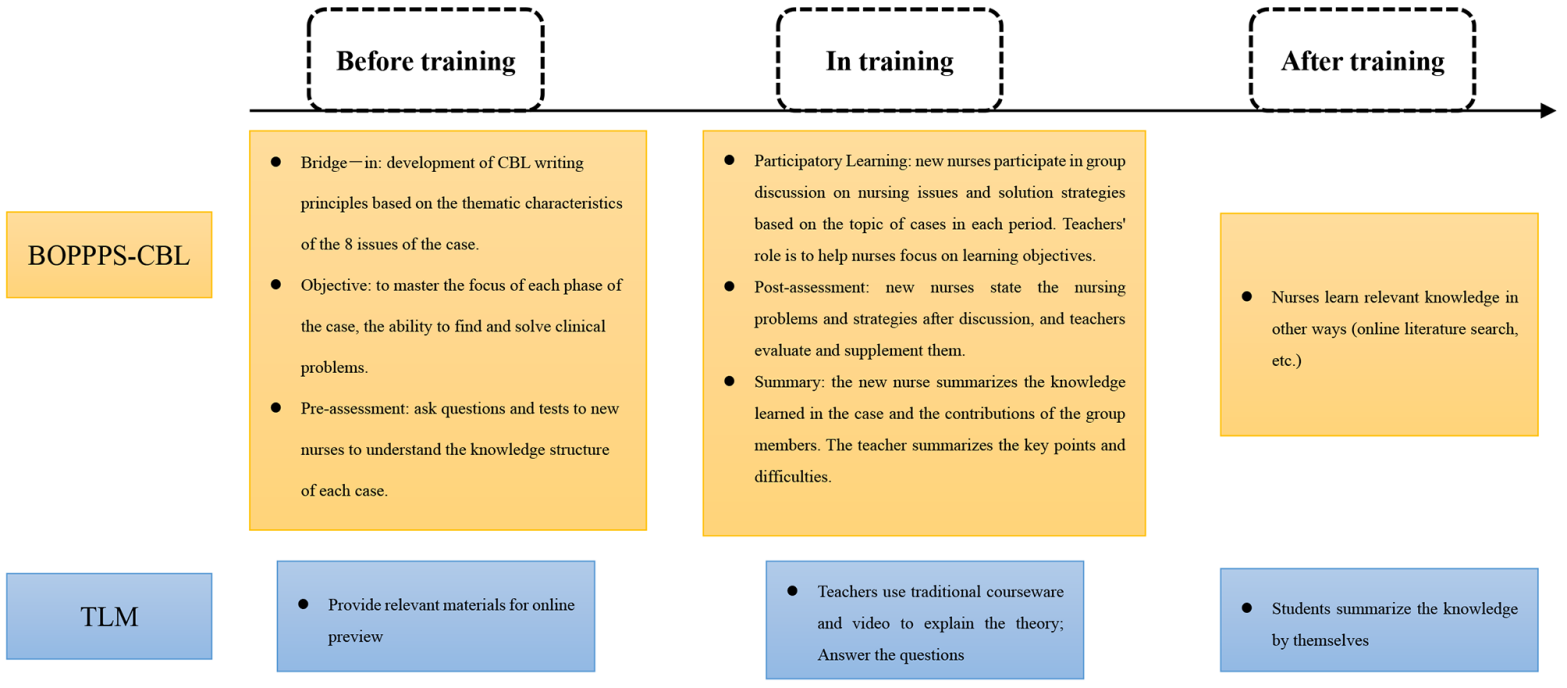
Flowchart of teaching design of the BOPPPS-CBL and TLM groups

Fifth, expert group meeting. According to Hasson’s opinion [31] the ideal number of experts selected is 4 ∼ 16, in this study 9 experts were selected for the panel meeting discussion, and the experts were chosen using the following criteria: (1) research areas covering nursing education (*n* = 4), medical education (*n* = 3), and psychology (*n* = 2); (2) familiarity with the field of nursing education research; (3) intermediate and higher professional technical titles plus a master’s degree or above; and (4) voluntary participation on this paper and provision of informed consent. The experts’ familiarity was Cs = 0.833; the experts’ judgment basis Ca = 0.957; and the experts’ authority coefficient Cr = 0.895: the training program was revised through the expert group meeting, and the final plan was developed. The general information about the experts, their level of familiarity, and the revisions proposed by the experts were shown in Appendix 1. The specific training mode was shown in Table 1. The BOPPPS-CBL and TLM model flowchart was summarized in Fig. 1.

### Data collection

#### The quantitative phase of the questionnaire survey

The Competency Inventory for Registered Nurses (CIRN) was a Chinese self-assessment instrument created by Liu [32] to assess nurses’ core competencies using the ICN’s Core Competency Framework for Nurses. In China, this scale is widely used by registered nurses. The CIRN includes 55 items organized into seven categories: critical thinking and research, clinical care, leadership, interpersonal relationships, ethical and legal practice, professional development, and educational consultation. The response options on a 5-point Likert scale ranged from 0 (not at all competent) to 4, with total scores ranging from 0 to 220. A higher score indicates greater core competency. It takes about 10 to 15 min to complete the inventory. The total Cronbach’s α coefficient was 0.89; each dimension was 0.718 to 0.908 and the Content Validity Index was 0.852. The total Cronbach’s α coefficient was verified in other studies as 0.92 − 0.76 [33]; indicating good reliability and validity.

The Theoretical Knowledge Examination was created by the research team to assess nurses’ mastery of theoretical knowledge after training. After content validity evaluation by 10 nursing teaching experts, the Scale-level Content Validity Index, S-CVI/AVe of the paper was 0.949. The Cronbach’s α was 0.822, and the difficulty level of the exam paper was medium. The questions were scored out of 100 points and included multiple-choice and short text-based questions.

#### Focus group interviews in the qualitative phase

To avoid causing stress to the interviewees, the hospital education service staff (with a certificate in qualitative research) conducted focus group interviews with the nurses in the intervention group. The interviews were conducted to gain an understanding of their experiences and suggestions for training, as well as to improve the BOPPPS-CBL training program. Purposive sampling was used to recruit intervention group participants, and before the interview, all participants were informed as to the aims of the study and volunteered to participate. At the end of the BOPPPS-CBL training, 24 nurses were divided into four focus groups (5–7 nurses per group) to participate in the interviews, of which 22 were women and 2 were men, aged between 20 and 26 years. The first draft of the interview outline was finalized through literature reading, research team, and expert group meeting discussions, and the final outline was discussed again after pre-interviews with four nurses. The following was included in the interview outline: (a) How do you feel about taking part in this BOPPPS-CBL training course? (b) How has your clinical work changed after this training? (c) In what areas of this training program do you believe improvements should be made, and why? Share your ideas and opinions with us. The location of our interviews was chosen to take place in a quiet classroom. Interviews lasted 45–55 min. The whole process was conducted according to the qualitative research method.

The researcher’s team directed the TLM and BOPPPS-CBL training. Before the implementation of the training program, 10 nursing teaching teachers and 3 nurses with master’s degrees in nursing were uniformly and systematically trained. We explained the training’s aim, the procedure, what to expect during implementation, and how to gather data to assure quality control.

### Data analysis

The quantitative data were coded and entered into IBM SPSS version 22 software for statistical analysis. Quantitative data for nurses were analyzed by t-test and expressed as mean ± standard deviation. Categorical data were analyzed by the chi-square test. The significance level for all tests was set at *p* = 0.05.

For the qualitative part, we transcribed the focus group data within 24 h after the interviews were completed, and the data were analyzed by YW and YC researchers (both of whom were certified in qualitative research), respectively, according to the Colaizzi Seven-Step Method [34], which includes: (1) transcribing the audio recordings into text promptly and reading them over and over again; (2) excerpting the statements that are closely related to the theme of the study; (3) coding the recurring and meaningful ideas; (4) pooling the coded ideas; (5) writing detailed and missing descriptions; (6) identifying similar ideas and sublimating the thematic concepts; and (7) returning the results to the interviewees for verification and validation. In cases of disagreement, a third researcher (XL) was involved. Considering the cultural nature of the language, our analyses were conducted after transcribing into Chinese and discussing it repeatedly, organizing, reading, summarizing, coding, and re-reading the data, and finally conducting a summary of the themes in Chinese, after which we translated the Chinese themes into English.

### Ethical considerations

The study protocol was approved by the Ethics Committee of Peking University People’s Hospital (2018THB145). Our study adhered to the principles of the Helsinki Declaration, and all participants provided their informed consent by signing a consent form. In the qualitative portion of the study, the research team members did not directly interact with the interviewees. Instead, we engaged a researcher from the hospital’s education office who informed the participants about the voluntary nature of their participation and assured them that there would be no negative consequences for their work. Additionally, the participants were informed that no personal information would be disclosed and that all data would be collected anonymously. The audio recordings of the interviews were transcribed within 24 h and subsequently destroyed within 3 weeks after obtaining confirmation from the interviewees.

## Results

### Demographic result

185 new nurses were enrolled in the trial, with the intervention group’s mean age at 21.97 ± 1.58 years and the control group’s mean age at 22.13 ± 1.63 years. A bachelor’s degree or higher had been earned by 47.1% of the intervention group and by 47.8% of the control group. The results indicated that there was no statistically significant difference (*P* > 0.05) between the intervention group and the control group when comparing general variables such as gender, educational level, and department. The baseline data of the two groups were comparable, as shown in Table 2.

**Table 2:** New nurse characteristics (*n* = 185)

Characteristics	BOPPPS-CBL group n(%)	TLM group n(%)	*t/X* ^*2*^	*p*
**Age(years) (mean (SD)**	22.13 ± 1.63	21.97 ± 1.58		
**Gender**			-0.928	0.328
Male	11(9.6)	4(5.7)		
Female	104(90.4)	66(94.3)		
**Education level**			-0.340	0.738
Junior college	60(52.2)	37(52.9)		
Undergraduate	48(41.7)	28(40)		
Master	7(6.1)	6(7.1)		
**Clinical Departments**			-0.260	0.796
Internal medicine	38(33)	22(31.4)		
Surgery	44(38.3)	27(38.6)		
Maternal and pediatrics	10(8.7)	6(8.6)		
Emergency / Severe	23(20)	15(21.4)		

SD: standard error; TLM: Traditional learning Mode; BOPPPS-CBL: BOPPPS model combined with case-based learning; *P*<0.05

### Quantitative results

There was no discernible difference between the two groups’ comparable CIRN results at the beginning of the training. Following 2 years of training, the BOPPPS-CBL group’s overall CIRN scores were compared with those of the TLM group, and the differences between the two groups’ CIRN scores were statistically significant (*t* = 8.240, *P* < 0.05). Additionally, the analysis of the two groups’ CIRN scores before and after the training revealed that both groups’ CIRN scores increased (TLM group: *t* = 5.661, *P* < 0.01; BOPPPS-CBL group: *t* = 7.148, *P* < 0.01) after the standardized training (Table 3).

**Table 3:** CIRN scores before and after training in the BOPPPS-LBL group and TLM group

Item	CIRN score before training ($\bar x \pm s$)	CIRN score after training ($\bar x \pm s$)	T-test	
			*t *value	*P* value
TLM group	148.84 ± 34.17	170.78 ± 21.30	-5.661	<0.001
BOPPPS-CBL group	157.51 ± 38.69	202.16 ± 30.44	-7.148	<0.001
*t* value	-1.591	-8.240		
*P* value	0.113	0.001		

CIRN: Competency Inventory for Registered Nurses; TLM: Traditional learning Mode;

BOPPPS-CBL: BOPPPS model combined with case-based learning; *P*<0.05

The theoretical knowledge examination scores of the BOPPPS-CBL group were significantly higher than those of the TLM group (79.36 ± 10.27 vs. 70.25 ± 9.31, *t* = 6.201, *P* < 0.01), and the difference was statistically significant (*P* < 0.05) (Table 4).

**Table 4:** Comparison of scores of theoretical knowledge examination between BOPPPS-CBL group and TLM group

Item	**theoretical knowledge examination scores** Mean ± standard	**T-test**	
		*t* value	*P* value
TLM group	70.25 ± 9.31	-6.201	0.001
BOPPPS-CBL group	79.36 ± 10.27		

TLM: Traditional learning Mode; BOPPPS-CBL: BOPPPS model combined with case-based learning; *P*<0.05

Comparing Table 3 with Table 5, the results indicate that the CIRN questionnaire comprised seven dimensions. Significant differences were observed between the two groups in the dimensions of critical thinking and research, clinical care, professional development, and educational consultation (*P* < 0.05). However, no differences were found in the dimensions of leadership, interpersonal relationships, ethical, or legal practice.

**Table 5:** Competency inventory for registered nurse’s total score and sub-dimension scores

Item	TLM group	BOPPPS-CBL group	*t/F* value	*p*
Critical thinking and scientific research	24.42 ± 5.76	34.56 ± 5.44	-11.847	<0.001
Clinical Nursing	23.43 ± 5.56	30.83 ± 4.93	-9.160	<0.001
Leadership	33.77 ± 3.78	34.57 ± 5.38	-1.182	0.239
Interpersonal relations	26.60 ± 4.71	27.87 ± 4.39	-1.826	0.069
Ethics and legal practice	27.18 ± 4.44	28.47 ± 4.83	-1.852	0.066
Professional development	16.62 ± 4.11	21.26 ± 3.5	-7.863	<0.001
Education consulting	18.75 ± 4.73	24.63 ± 4.04	-8.658	<0.001

TLM: Traditional learning Mode; BOPPPS-CBL: BOPPPS model combined with case-based learning; *P*<0.05

### Qualitative results

Twenty-four nurses participated in focus group interviews to investigate their experiences with BOPPPS-CBL training. The resultant data was analyzed, and three major themes and six subthemes were identified. The first theme, role facilitation, was characterized by the stimulation of interest in learning, affirmation of the benefits of the training, and clarification of the orientation of clinical nurses. The second theme, forming new thinking about solving clinical problems, encompassed learning to analyze clinical issues from diverse perspectives, improving communication skills, expanding cognitive abilities, and fostering teamwork. The third theme focused on suggestions for improvement (Table 6).

**Table 6:** Qualitative focus group

Categories	Subcategories	Quotations
Role facilitation	Stimulate interest in learning and affirm the benefits of training	*“Group peer discussion was very interesting and stressful, we have to check more and ask more questions and communicate more, otherwise it would be embarrassing to wait until the time of reporting”N6* *“I like this kind of training, it makes me more involved and the experience is very good” N2*
	Clarify clinical nurse orientation	*“… It broke my previous perception that clinical nurses just listen to doctors and give injections…”N23* *“I now understand that as a qualified clinical nurse, I should be able to take the initiative to explore, solve problems, and think critically…”N15*
Form new thinking to solve clinical problems	Learn to analyze clinical problems from multiple perspectives	*“I will think on my own first, organize into different modules, and then check the information on the Internet, also will turn to books…”N5* *“Now I will seek advice from experienced nurses and bedside doctors”N4* *“The biggest difference is that I now put the patient’s needs first, and then formulate nursing care measures based on this”N13*
	Enhanced communication skills	*“Through group discussions, I can clearly express my own views”N19* *“I don’t resist communicating with patients and doctors as much as I used to, and I even enjoy it a little bit”N5*
	open up thinkin	*“After the training, I have learned from different partners how they look at problems”N1* *“I especially like the teacher’s feedback at the end, I can know where there are deficiencies, and next time I encounter similar problems, I can learn from them”N7* *“Now when I encounter a problem, I will ask myself why and what to do, and then analyze it with a mind map”N3*
	Promoting teamwork	*“I really enjoyed the learning atmosphere. We actively discuss typical cases, divide up the work…”N8* *“It was so much fun to work together. We clarified our goals and then supported each other to solve problems together”N19*
suggestions for improvement		*I hope that there will be a visual simulation in the future, so that I can really immerse myself in the case scenarios!N6* *If these materials can be recorded and put online for us to watch over and over again, the learning effect will be very good! N20*

Of the nurses interviewed, 87.6% (21/24) expressed that the BOPPPS-CBL training had numerous benefits, asserting that the training enhanced their understanding of the role and orientation of clinical nurses. Each participant played a fundamental role in constructing knowledge during case study processing, which focused on self-directed learning and developed problem-solving skills in nursing practice through multidimensional analysis and discussion of a range of cases. Furthermore, participants reported that group peer discussions facilitated learning, enhanced their enjoyment of inquiry-based self-directed learning, and increased engagement.

Respondents also conveyed enhanced confidence and proficiency in adopting proactive communication practices, examining clinical issues from diverse viewpoints, and thinking innovatively. These acquired skills were subsequently applied in fostering effective communication between healthcare professionals and patients, influencing patient clinical decision-making, and optimizing care delivery. Participants affirmed that the BOPPPS-CBL training played a pivotal role in amalgamating theoretical medical knowledge with practical clinical care, thereby bridging the gap between the two domains. It facilitated the cultivation of a novel perspective on clinical challenges and equipped them with the ability to utilize mind maps for problem analysis and clinical decision-making. This integrative approach not only instilled a sense of accomplishment but also underscored the value they contributed to their clinical practice.

However, other nurses suggested ideas to improve the training, such as providing more teaching resources and adopting deeper teaching approaches. They also expressed hope that visualization technologies could be utilized in the future to create immersive settings, while online learning resources could be made available for trainees to watch and learn from repeatedly.

## Discussion

In this study, an explanatory mixed-methods approach was employed to analyze quantitative and qualitative data to validate the effectiveness of BOPPPS-CBL training. The quantitative and qualitative results were complementary to each other. In the first part of the study, the quantitative research supported the research hypothesis that BOPPPS-CBL training was effective in improving the core competencies and theoretical knowledge scores of newly recruited nurses. In the second part of the study, the qualitative study affirmed the benefits of BOPPPS-CBL on the clinical roles and thinking skills of newly recruited nurses, which increased their enthusiasm and enjoyment of independent learning and affirmed the effectiveness of the training method.

In the first part of the study, we examined alterations in the core competencies of nurses before and after their participation in the TLM and BOPPPS-CBL groups. Both groups exhibited an elevation in CIRN scores post-training, indicating that both training programs enhanced nurses’ competencies, which was consistent with Burgess’ findings [35]. However, the quantitative study revealed that the training in the BOPPPS-CBL group was particularly effective, leading to a greater enhancement in nurses’ core competencies and theoretical knowledge compared to the TLM group. This difference can be attributed to several factors. First, BOPPPS was grounded in constructivist and humanistic learning theories [23], while CBL constituted an application of social cognitive theory [36]. BOPPPS-CBL, as an innovative medical teaching approach, emphasized the autonomy of nurses’ learning abilities, prioritized nurse participation and feedback, and facilitated the transformation of nurses from passive recipients of external stimuli and recipients of indoctrination to active information processors and meaning constructors. This transformative process encouraged the mastery, internalization, and absorption of knowledge. These outcomes align with the findings of Xue et al.‘s research [37]. In contrast, the TLM model, being teacher-centered and heavily reliant on the curriculum, led to passive knowledge reception by students with limited autonomous learning capabilities [38].

Moreover, in the BOPPPS-CBL training, the learning process was compartmentalized into organic modules through the application of BOPPPS, with each module thoroughly engaging and motivating the nurse [23]. Simultaneously incorporating the benefits of CBL, the training proceeded by building on real-life cases. In Bridge-in, to attract the nurses’ attention and stimulate their interest, the objective was to make it clear the goal of this learning and the direction of teaching. In the BOPPPS-CBL training, participatory learning, repeated exchanges and collisions among peers, grounded in the case and their individual knowledge, enhanced nurses’ initiative, knowledge acquisition, and core competencies. These outcomes align with the findings of previous studies [39].

In the second phase of the study, we analyzed nurses’ experiences with participating in BOPPPS-CBL training. Most nurses affirmed the effectiveness of BOPPPS-CBL training. Nurses indicated that various forms of CBL methods heightened their enjoyment of learning, fostered a positive collaborative learning atmosphere, and enhanced their comprehension abilities. Consistent with previous research, diverse learning formats increase student engagement and intrinsic motivation [40]. In the current study, nurses provided feedback on encountering real-life cases from clinical practice, which conveyed responsibility and pressure, compelling them to clarify their roles as competent clinical nurses. Previous research has indicated that feedback from teachers contributes to student growth [41]. In our study, we discovered that, in addition to teacher feedback helping nurses identify knowledge construction issues when dealing with clinical problems, discussions among peers also expanded nurses’ thinking, allowed them to draw on their peers’ strengths, and aided in their personal development. This may be related to the philosophical concept of “self-cultivation” advocated in Chinese Confucian culture, promoting a learning attitude that emphasizes humility, continuous learning from others, and avoiding arrogance and impatience [42]. Interestingly, nurses also shared changes in their clinical thinking as BOPPPS-CBL training challenged their inherent learning and thinking patterns, such as rote learning, passive knowledge acceptance, and memorization. This stimulation prompted them to actively explore and analyze clinical issues from multiple perspectives, enhancing their ability to collaborate and communicate with healthcare professionals, patients, and peers in problem-solving. This may be linked to the Confucian cultural background, where the “middle ground” principle in education emphasizes the cultivation of positive interpersonal relationships and the importance of collaborative cooperation [43]. In addition, 87.65% of the nurses in the focus groups indicated that they strongly preferred and supported the promotion of the BOPPPS-CBL training model, which was consistent with previous studies [39]. However, some nurses also indicated that if future BOPPPS-CBL training could leverage intelligent visual aids, immersive scenario simulations, and additional online learning resources, facilitating repeated viewing after training, it would further enhance their ability to apply the learning to clinical practice.

Our research findings indicate that quantitative research confirmed the efficiency of BOPPPS-CBL training, while qualitative research investigated the fundamental variables and underlying motivations that contribute to its effectiveness. The qualitative findings supplemented and confirmed the benefits of BOPPPS-CBL training revealed in the quantitative analysis. The training, which was based on the BOPPPS closed-loop instructional process model with nurses at its core, grounded in real-life experiences, and guided through group discussions, proved effective in immersing nurses in clinical environments. This approach facilitated the cultivation of clinical practice skills and the learning of core competencies, ultimately benefiting newly recruited nurses.

### Limitations and implications

This was the first time that BOPPPS-CBL was used in China in the education and training of new nurses. This study design combines the capabilities of quantitative and qualitative research by first evaluating the effectiveness of comparing TLM and BOPPPS-CBL through a quantitative research design and then refining it through interviews with nurses in the BOPPPS-CBL group. The findings of this study demonstrate that it was successful and beneficial. Our study, however, had several limitations. Firstly, because this was a classroom experiment, it was impossible to control all of the confounding factors of the training effect, especially when both the intervention and control groups were training. Given the 2-year training interval and the intervention group training during the COVID-19 epidemic, one must consider the influence of external environmental factors arising from the epidemic on the results. Secondly, the predominant inclusion of female subjects raises uncertainty about the generalizability of the results to the male nurse population in China. Thirdly, we did not compare the differences between the BOPPPS model and CBL, a consideration that could be addressed in a future study comparing BOPPPS, CBL, and TLM. Moreover, our investigation into the effect on nurses’ core competencies relied on self-assessment questionnaires, which, to some extent, might not truly and objectively reflect nurses’ core competencies. In the future, utilizing an objective assessment tool could provide a more accurate evaluation of the efficacy of BOPPPS-CBL training in enhancing nurses’ core competencies. Fourthly, this study only examined the effects at the conclusion of the standardized training for new nurses, without assessing the long-term effects. Longitudinal studies based on BOPPPS-CBL training programs could be conducted in the future to explore how the core competencies developed by nurses manifest in long-term clinical practice. Finally, this study exclusively focused on the effect within one hospital; future large-scale multicenter validation studies could be undertaken in different regions and hospital levels.

## Conclusions

This study showed that the BOPPPS-CBL model was more effective than TLM, and the core competencies and theoretical knowledge of nurses in the BOPPPS-CBL group increased significantly. Focus group nurses also confirmed the benefits of BOPPPS-CBL training in terms of role enhancement and clinical decision-making thinking. The BOPPPS-CBL model is an effective pedagogical model for the standardized training and education of new nurses. More research in multicenter studies incorporating smart teaching tools is needed to validate the effectiveness of the model in other contexts. In addition, the model may provide new ideas for researchers or clinical education administrators in other countries when developing continuing education training programs for nurses.

### Electronic supplementary material

Below is the link to the electronic supplementary material.

Supplementary Material 1

## Data Availability

Our research data is related to the personal identity information of nurses. If the data sets analyzed during the study are used publicly, there is a risk of the personal privacy disclosure of nurses. Therefore, we declare that the data will not be disclosed. If there is a strong demand, please send a request to the corresponding author (XD L, lxd_2000_510@163.com).
