# Supplementary material for: Assessment of the effectiveness of the BOPPPS model combined with case-based learning on nursing residency education for newly recruited nurses in China: a mixed methods study
Source: BMC Med Educ. 2024 Mar 1;24:215. doi: 10.1186/s12909-024-05202-x (PMC10908075; doi:10.1186/s12909-024-05202-x)
Supplement: Supplementary file 1 — Supplementary Material 1 [file 12909_2024_5202_MOESM1_ESM.docx]

**Appendix 1: Includes the general information of experts, the authority coefficient of experts, the judgment basis of experts**

Appendix table 1 General Information of consulting experts (*n*=9)

| No | Age（yesrs） | | Field of work | Job title | Education level | | Years of work | |
| --- | --- | --- | --- | --- | --- | --- | --- | --- |
| 1 | | 46 | Psychology | Professors | | Ph.D | | 22 |
| 2 | | 42 | Psychology | Associate Professor | | Ph.D | | 21 |
| 3 | | 45 | Nursing Education | Director | | Ph.D | | 14 |
| 4 | | 40 | Nursing Education | nurse deputy director | | Ph.D | | 12 |
| 5 | | 51 | Nursing Education | Director | | bachelor's degree | | 33 |
| 6 | | 47 | Nursing Education | Director | | Ph.D | | 25 |
| 7 | | 45 | Medical Education | deputy chief physician | | Ph.D | | 18 |
| 8 | | 55 | Medical Education | Chief Physician | | Ph.D | | 30 |
| 9 | | 49 | Medical Education | Chief Physician | | bachelor's degree | | 26 |

Appendix table 2 Results of self-assessment of expert familiarity

|  | Know well | familiar | General familiarity | Not very familiar | Very  unfamiliar |
| --- | --- | --- | --- | --- | --- |
| Point value | 0.9 | 0.7 | 0.5 | 0.3 | 0.1 |
| Number of  Experts | 6 | 3 | 0 | 0 | 0 |

Cs=∑MjWj/M= (6×0.9+3×0.7) ÷9=0.833

Appendix
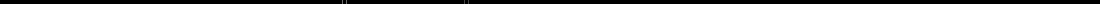
table 3 The basis of expert judgment and the relevant scores of the influence degree of expert judgment

Basis of Expert Judgment

|  | big | center | small |
| --- | --- | --- | --- |
| Practical experience | 7 (0.5) | 2 (0.4) | 0 (0.3) |
| Theoretical analysis | 7 (0.3) | 2 (0.2) | 0 (0.1) |
| Get to know your peers | 6 (0.1) | 2 (0.1) | 1 (0.1) |
| Intuitive intuition | 0 (0.1) | 2 (0.1) | 7 (0.1) |

A score for the degree of influence on expert judgment

Ca= ∑M_j_W_j_/M _=_(7×0.5+7×0.3+6×0.1+2×0.4+2×0.2+2×0.1+2×0.1+1×0.1+7×0.1)÷9=0.957

Appendix table 4 Summary of expert opinions

| Item | Existing issues | revision of opinion |
| --- | --- | --- |
| Training objective | The training objectives are not clea | Establish short-term and long-term clear training goals |
| Training time | Each training session lasts 4 hours | If the time is too long, the concentration will be lost, it is recommended to take 3h each time and take a 20min rest every 1.5h |
| Training content | Difficulty of case training cannot be consistent | For the first 2 trainings, the cases should be relatively simple to familiarize the nurses with the process and considerations of the training program, followed by taking ownership of the new nurses. |
| Evaluation of Training | Using only quantitative methods to evaluate the effectiveness of training programs | It is recommended that focus group interviews be added to understand the benefits and shortcomings of the training programme and follow up to optimize the programme again |
| Evaluation of Training | Team of researchers lead focus group interviews | Suggested to avoid stressing respondents, staff in hospital education services (with qualitative research certificates) |
